# Upward spontaneous migration of ventriculoperitoneal shunt into the heart: A case report summary

**DOI:** 10.34172/jcvtr.2022.30523

**Published:** 2022-12-31

**Authors:** Shamsi Ghaffari, Khosro Hashemzadeh, Mahmood Samadi, Akbar Molaei, Sahar Sadeghi, Ahmad Jamei Khosroshahi

**Affiliations:** ^1^Cardiovascular Research Center, Tabriz University of Medical Sciences, Tabriz, Iran; ^2^Pediatric Research Center, Tabriz University of Medical Sciences, Tabriz, Iran

**Keywords:** Hydrocephalus, Ventriculoperitoneal, Shunt Migration, Mediastinum

## Abstract

A male infant with a history of ventriculoperitoneal (VP) implantation due to congenital hydrocephalus presented with fever and lethargy at the age of 8 month-old. Pericardial effusion was detected in transthoracic echocardiography, and he underwent pericardial window operation and was discharged in a stable condition. At 11 months of age, he presented again with fever, lethargy, recurrent vomiting, and respiratory distress. In both plain chest radiography and transthoracic echocardiography, VP shunt migration to the heart cavity was observed. The VP shunt had entered into the right ventricle after perforating the diaphragm and pericardium. The patient underwent open-heart surgery due to vegetation at the tip of the VP shunt inside the right heart. Vegetation was removed and the tip of the shunt was returned to the peritoneal cavity. Two weeks after discharge, the patient presented again with symptoms of tachypnea and lethargy. The imaging revealed the entry of the VP shunt about two centimeters into the anterior mediastinum. The patient was transferred to the operation room and the VP shunt was shortened and re-inserted into the peritoneal cavity. Antibiotic treatment was continued for six weeks and the patient was discharged in stable condition. In follow-up visits after two years, the VP shunt functioned well and no particular complication was observed. This case demonstrates that in patients with VP shunt implantation presenting with pulmonary and cardiac symptoms such as respiratory distress, pericardial effusion, and cardiac tamponade after VP shunt implantation, the possibility of VP shunt catheter migration to the mediastinal cavity should be considered.

## Introduction

 Hydrocephalus is a heterogeneous disease and may affect different age groups. In an infant, with no obvious extrinsic cause, hydrocephalous is usually referred to as congenital. Condition such as intracranial hemorrhage, infection, or tumors may lead to acquired or secondary hydrocephalus. In contrast to its complex etiology, limited therapeutic options are available.

 Ventriculoperitoneal (VP) shunt implementation is a common procedure in neurosurgery which is mainly used for the treatment of hydrocephalus and is performed approximately on 30 000 patients each year in the United States. Several complications, including hemorrhage, cerebrospinal fluid (CSF) pseudocyst formation, infection, hypersensitivity reaction or intolerance and blockage had been reported. Migration of distal end of catheter into thorax, mediastinum, heart, stomach, bladder, and scrotum are among the reported complications.^1^

 In this paper, we report a case of upward distal migration of the VP shunt to the mediastinal cavity, and heart perforation in an infant suffering from hydrocephalous. The case is reported after obtaining the permission of the institutional review board.

## Case Presentation

**Figure 1:**
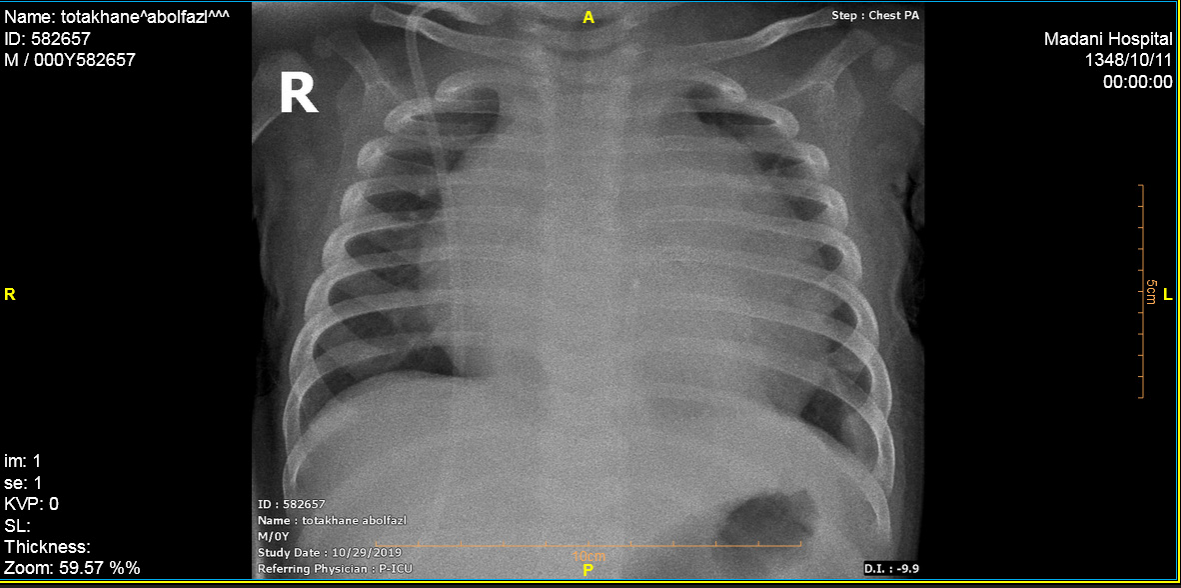
Plain chest radiography depicting pericardial effusion (at the age of 8 month old).

**Figure 2:**
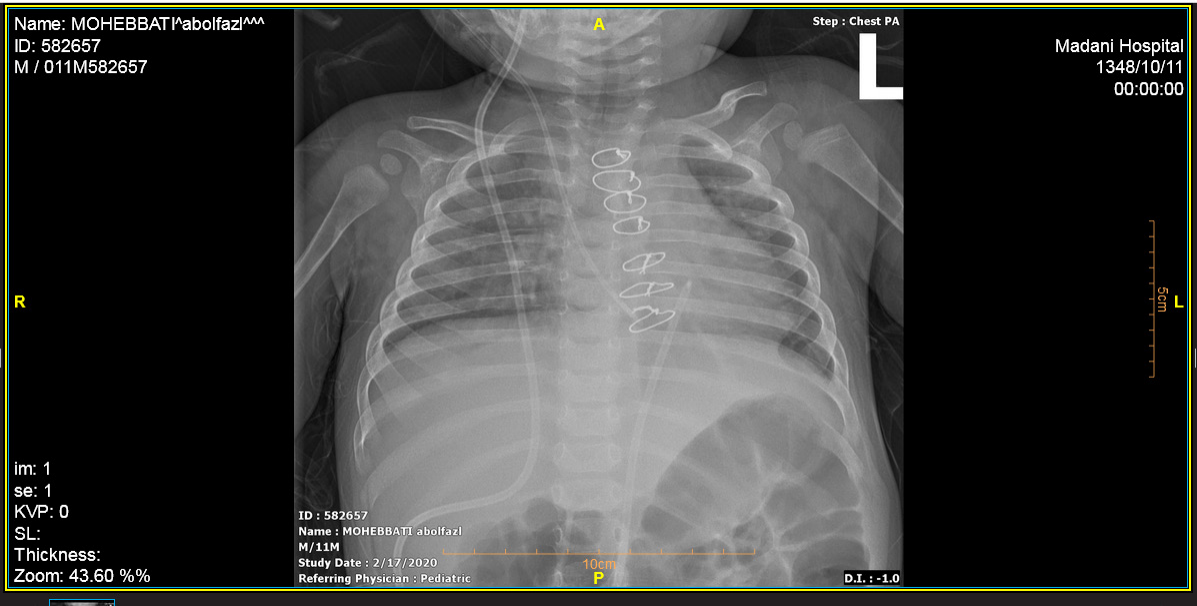
Plain chest radiography depicting the entrance of VP shunt into the heart cavity (at the age of 11 month old).

 A male infant who underwent VP implantation due to congenital hydrocephalus at the age of 2-month-old presented with fever and lethargy at the age of 8-month-old. Pericardial effusion was detected in transthoracic echocardiography and plain chest radiography (Figure 1), and he underwent pericardial window under general anesthesia and a mediastinal chest drain was implanted in the pericardial space. After draining the excessive pericardial fluid, the mediastinal chest drain was removed and the patient was discharged under appropriate conditions. The patient presented again at 11 months of age due to fever, lethargy, recurrent vomiting, and respiratory distress. In chest radiography migration of the distal end of VP catheter into mediastinum was suspected. More evaluation by transthoracic echocardiography (TTE) showed the entrance of catheter into right ventricle after perforating the diaphragm and pericardium. Meanwhile a mass lesion at tip of catheter was detected in favor of vegetation and endocarditis (Figure 2).

**Figure 3:**
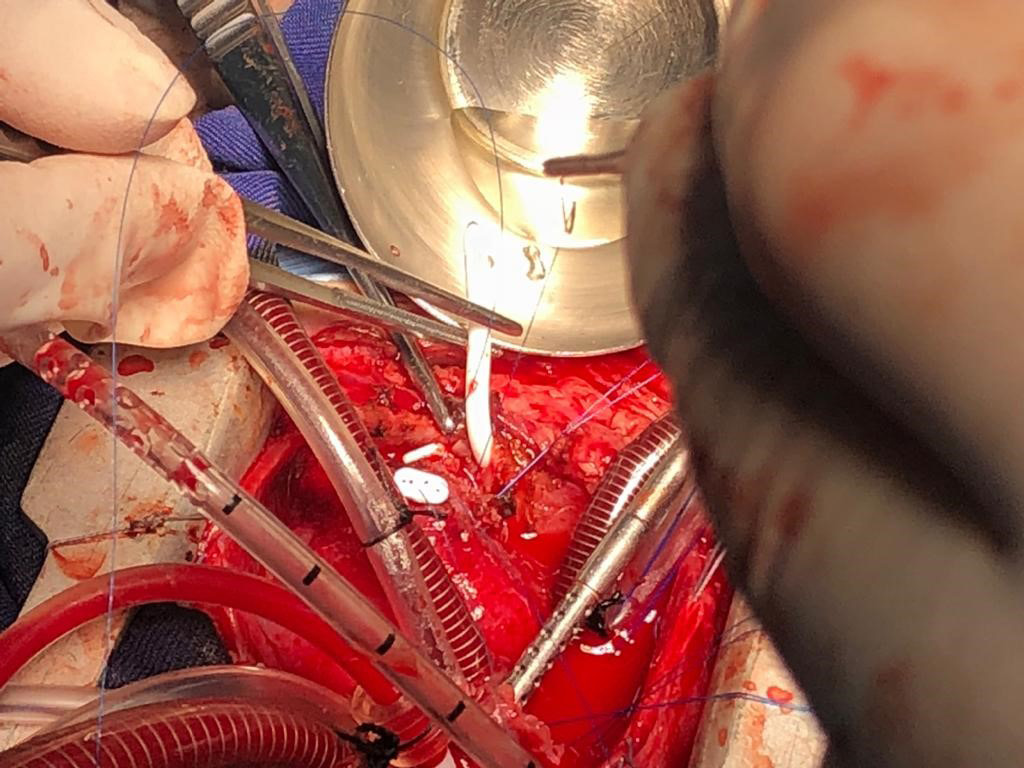
The patient underwent open heart surgery due to vegetation at the tip of VP shunt inside the right ventricle.

**Figure 4:**
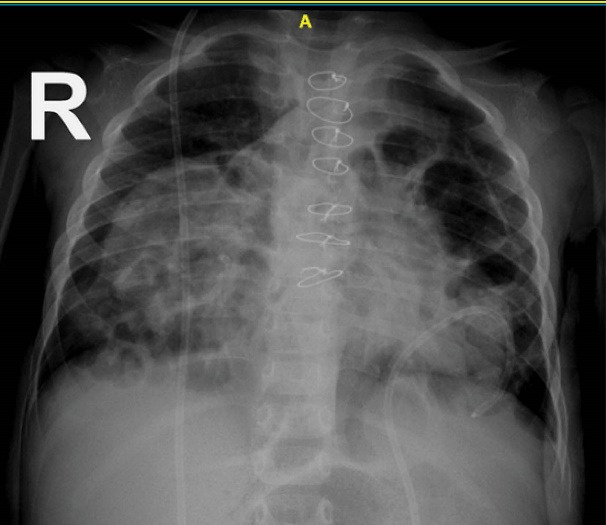
Plain chest radiography depicting re-entrance of VP shunt into the anterior mediastinum.

**Figure 5:**
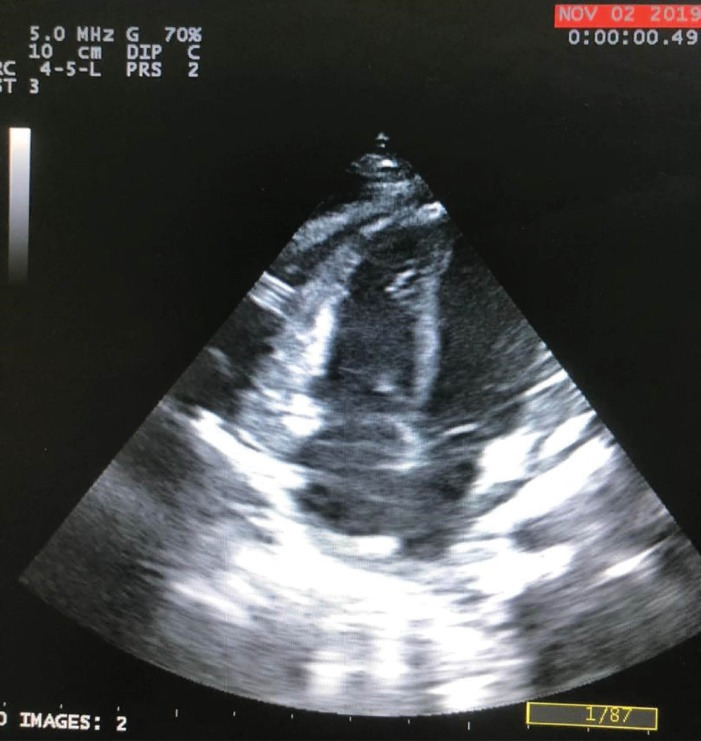
Echocardiography image: White arrow shows catheter tip.

 The patient planed for open-heart surgery and all the necessary arrangements were made.(Figure 3). Cardiopulmonary bypass was conducted under general anesthesia with a middle sternotomy technique and cannulation of the superior and inferior vena cava. The body was cooled to 30 °C and the cardiopulmonary solution was injected from the root of the heart aorta. In the right ventricle, a vegetation of 5 cm long was observed originating from the entrance point of the VP shunt and continuing up to the right ventricular outflow tract and the tricuspid valve. The vegetation was extracted and the tip of the VP shunt was directed back to the peritoneal cavity. The tricuspid valve was intact with appropriate function. The right ventricle and atrium were repaired and the patient was detached from cardiopulmonary bypass after warming up. Two drains were placed in the pleural space and one drain in the mediastinum and the sternum was closed. The patient was transferred to the intensive care unit (ICU) in stable condition and recovered after one day. Two weeks later, the patient was discharged in stable condition. Two weeks after discharge, the patient presented again with symptoms of vomiting, tachypnea, and lethargy. Chest X-ray and thoracic echocardiography revealed that the VP shunt was again migrated about 2 cm into the anterior mediastinum (Figures 4 and 5). The patient underwent reoperation under general anesthesia and the VP shunt was shortened and re-inserted into the peritoneal cavity. The patient was transferred to the ICU in stable condition and recovered after 6 hours. Antibiotic treatment was continued for six weeks and the patient was discharged in stable condition. In follow-up visits after two years, the VP shunt functioned well and no particular complication was observed.

## Discussion

 Ultrasonography as non- invasive, radiation free, and portable modality helped us for initial brain and cardiac evaluation of our patient. Using transfontanelle cranial ultrasonography we found no specific lesion such as vein of Galen malformation (arteriovenous malformation), periventricular leukomalacia, Chiari malformation, Dandy-Walker complex, prenatal infections, and tumors as a cause for hydrocephalus. Bed side initial cardiac evaluation by echocardiography confirmed no associated cardiac defect, and tumor.^2^ Determining a management plan, we used brain CT scan and despite predictable complications, insertion of VP shunt was inevitable due to progressive hydrosephalus.

 In 55% of cases of VP shunt implementation, revisions are unavoidable and usually occur three months after implantation.^3^ The main symptoms predicting VP shunt dysfunction have been reported to be headache, gait imbalance, cognitive impairment, and urinary incontinence.^4^ The causes of VP shunt complications are multifactorial but can be mainly divided into three groups: 1) infection, 2) mechanical failure and 3) functional failure.^5^ VP shunt migration is one of the rare complications of mechanical failure. The currently available reports in the literature have identified different destinations for migration of the distal part of the shunt catheter such as umbilicus, colon, stomach, bladder, scrotum, anus, thorax, and heart. Shunt migration to the mediastinal cavity can be classified as supradiaphragmatic (there is no part of the shunt below the diaphragm) and trans-diaphragmatic (in which the shunt passes from below the diaphragm to the thoracic cavity). Spontaneous entry of the VP shunt into the heart cavity is one of the rare cases of shunt migration and has been documented in a few studies and our report.^6^ The slow movement of the shunt into the thorax due to negative intrathoracic pressure following respiration is one of the possible mechanisms of VP shunt migration to the chest. Other possible mechanisms include migration due to an intra-abdominal inflammatory response or entry through a congenital hernia or perforation in the diaphragm. Shunt migration is associated with age and is more common in early childhood and the elderly, which can be attributed to muscle and vascular weakness in this age range.^7^

 Kanojia et al reported that distal VP shunt migration accounted for approximately 10% of all VP shunt complications.^8^ Moreover, in a review by Harischandra et al cardiovascular migration accounted for 7% of all VP shunt migrations.^9^ Catheter entry into the heart cavity and pericardial space due to complications such as cardiac outflow obstruction, arrhythmia, clot embolism, pericardial effusion, and subsequent cardiac tamponade causes hemodynamic instability and is characterized as a life-threatening condition. In the study of Wiwattanadittakul et al the occurrence of cardiac tamponade and pericardial effusion was also reported due to the discharge of VP secretions into the mediastinal cavity following the migration of VP shunts into the heart cavity, which reduced pulse pressure and instability of vital signs and finally, the patient’s condition was stabilized by pericardiocentesis and catheter removal.^10^ In a similar study, Karakurt et al reported the occurrence of cardiac tamponade and pericardial effusion following the migration of the VP shunt into the pericardial space, which was resolved by pericardiotomy and repositioning of the shunt catheter in the abdominal space after shortening.^11^

 VP shunt migration to the heart can occur through a variety of pathways, including the venous system and the internal jugular vein. Zairi et al reported a 63-year-old patient with VP shunt migration into the pulmonary artery by perforating the collateral vein of the right internal jugular vein through the right ventricle of the heart.^12^ Kano et al also reported the migration of the VP shunt catheter into the internal jugular vein and right atrium, in which the catheter was removed with a small incision in the subcutaneous area and fixed in the abdomen.^13^ Ruggiero et al postulated that the possible cause of VP shunt catheter migration through the internal jugular vein is the result of direct damage to the arteries during tunneling.^14^ Moreover, negative intrathoracic pressure and venous flow together augment the possibility of catheter migration into the heart.

 Numerous methods for removing the migrated VP shunt have been described in the literature, which varies according to the patient’s condition. If there is a scar, clot, or vegetation around the catheter, surgery will likely be needed to remove it. In our study, due to the presence of vegetation at the distal end of the catheter and fixation of the catheter to the inner wall of the right ventricle, an operation was conducted to remove the VP shunt catheter. Ralston et al reported a case in which a VP shunt was implanted at the age of 7 years old due to hydrocephalus, but at the age of 17 years old he presented with a distal shunt catheter migration into the heart and pulmonary arteries. Following the formation of scars and clots around the catheter due to delayed presentation, the catheter was relatively fixed, which eventually required open-heart surgery to remove the catheter.^15^ In another case presented by Lancini et al VP shunt catheter had entered into the heart cavity through the jugular vein and superior vena cava and after making a loop into the main pulmonary artery via the right heart, the distal shunt tip crossed the patent foramen ovale to lodge in the left upper pulmonary vein. Inevitably the VP shunt was removed through atriotomy due to the presence of clots and fibrinous tissue.^16^ Finneran et al also reported a case of VP shunt implementation in an 81-year-old man whose distal shunt had subsequently entered into the right and left atria through the superior vena cava, and therefore by means of interventional radiology and open surgery the distal catheter was placed in the abdomen.^7^

 By reason of the high complication rates and potential risks of open surgery, it is considered to be the last option for removing a displaced VP shunt. Successful outcomes have been reported by conducting an endovascular approach for extraction of displaced VP shunt.^17^ Nguyen et al reported the extraction of VP shunt by endovascular technique no major complication despite the existence of knot of the distal end of the catheter in a patient with a displaced VP catheter in the right branch of the pulmonary artery.^18^ Nevertheless, some challenges can be faced in this technique. Chong et al reported upward migration of a VP shunt catheter into the right ventricle. They tried to remove the catheter by fluoroscopy through the internal jugular vein, which was failed due to kinks and knots of the distal end of the catheter. Therefore, they inevitably performed the right femoral vein puncture and the VP shunt catheter removed using a snare catheter.^19^

## Conclusion

 In patients with VP shunt implantation presenting with pulmonary and cardiac symptoms such as respiratory distress, pericardial effusion, and cardiac tamponade after VP shunt implantation, the possibility of VP shunt catheter migration to the mediastinal cavity should be considered. Therefore, periodic radiographic examination of the correct function of the VP shunt and its correct location is essential even in the absence of signs of malfunction. Nevertheless, considering the radiation risks the optimum time interval between periodic radiographic examinations should be evaluated in future cohort studies.

## Acknowledgements

 Authors would like to thank the operating room of the children’s hospital, Tabriz, Iran and the surgeons who participated in the patient’s surgery.

## Author Contributions

**Conceptualization:** Shamsi Ghaffari.

**Methodology:** All authors.

**Validation:** All authors contribute to validate the work.

**Investigation: **Shamsi Ghaffari, Khosro Hashemzadeh.

**Resources:** All authors.

**Data Curation:** All authors.

**Writing—Original Draft Preparation:** Shamsi Ghaffari, Sahar Sadeghi.

**Writing—Review and Editing:** Shamsi Ghaffari, Mahmood Samadi, Akbar Molaei.

**Visualization:** Akbar Molaei.

**Supervision:** Ahmad Jamei Khosroshahi.

**Project Administration:** Shamsi Ghaffari.

**Funding Acquisition:** All authors.

## Funding

 This project is financially supported by the author.

## Ethical Approval

 Informed consent was obtained from the parents of the patient.

## Competing Interests

 The authors declare that they have no conflict of interest.
